# Robotic Complete Mesocolic Excision (CME) is a safe and feasible option for right colonic cancers: short and midterm results from a single-centre experience

**DOI:** 10.1007/s00464-020-08194-z

**Published:** 2021-01-05

**Authors:** Najaf Siddiqi, Samuel Stefan, Ravish Jootun, Ioannis Mykoniatis, Karen Flashman, Richard Beable, Gerald David, Jim Khan

**Affiliations:** 1 Department of Colorectal Surgery, Portsmouth Hospitals NHS Trust, Queen Alexandra Hospital Portsmouth, Portsmouth, UK; 2 University of Portsmouth, Portsmouth, UK

**Keywords:** Robotic surgery, Complete Mesocolic Excision, Right colon cancer, Techniques

## Abstract

**Background:**

Complete mesocolic excision (CME) for right colon cancers has traditionally been an open procedure. Surgical adoption of minimal access CME remains limited due to the technical challenges, training gaps and lack of level-1 data for proven benefits. Currently there is limited published data regarding the clinical results with the use of robotic CME surgery. **Aim** To report our experience, results and techniques, highlighting a clinical and oncological results and midterm oncological outcomes for robotic CME.

**Aim:**

To report our experience, results and techniques, highlighting a clinical and oncological results and midterm oncological outcomes for robotic CME.

**Methods:**

All patients undergoing standardised robotic CME technique with SMV first approach between January 2015 and September 2019 were included in this retrospective review of a prospectively collected database. Patient demographics, operative data and clinical and oncological outcomes were recorded.

**Results:**

Seventy-seven robotic CME resections for right colonic cancers were performed over a 4-year period. Median operative time was 180 (128–454) min and perioperative blood loss was 10 (10–50) ml. There were 25 patients who had previous abdominal surgery. Median postoperative hospital stay was 5 (3–18) days. There was no conversion to open surgery in this series. Median lymph node count was 30 (10–60). Three (4%) patients had R1 resection. There was one (1%) local recurrence in stage III disease and 4(5%) distal recurrence in stage II and stage III. There was no 30- or 90-day mortality. Three-year disease-free survival was 100%, 91.7% and 92% for stages I, II and III, respectively. Overall survival was 94%.

**Conclusions:**

Robotic CME is feasible, effective and safe. Good oncological results and improved survival are seen in this cohort of patients with a standardised approach to robotic CME.

Minimally invasive colorectal surgery has undergone an exponential growth in recent years with studies demonstrating its feasibility, safety and oncological efficiency [1–3]. Total mesorectal excision (TME) has been the gold standard surgical approach for rectal cancers and has shown reduced local recurrence and improved survival [4–6]. While advances have been made in early detection, radiological staging, and surgical techniques resulting in improved outcomes for rectal cancer patients, the same has not been the case for colon cancers. Late presentation, tumour biology and variations in surgical approaches have been some of the factors responsible for poor outcomes in right colon cancers [7].

The concept of complete mesocolic excision (CME) popularised by Werner Hohenberger [8] is based on the principles that underpin TME surgery and can be applied to colon cancers too with a central vascular ligation and excision of complete envelope of the mesocolon. The early experience of CME surgery comes from open procedures. Minimal access approaches have been applied in selected cases; however, there is no consensus on the standardised technique [9]. In literature, different techniques and approaches for CME surgery (open, laparoscopic and robotic) have been published, including, top-down [10, 11], bottom-up [12] and SMV first approach [13]. There is no level-1 evidence to support the superiority of laparoscopic or robotic approaches and at present there are no published guidelines. Robotic surgery offers a dextrous and stable operating platform with the added benefit of wristed instruments and 3D vision make this challenging task safer to perform [14]. Anatomical variations, close proximity of important structures and unfamiliarity with the surgical planes have deterred many surgeons from adopting this technique in their routine use. There are also concerns about the morbidity associated with CME particularly in terms of vascular injury, prolonged ileus and lymphatic leak [15].

## Need for CME surgery

Regardless of the approach, CME surgery has been shown to be feasible and safe in many published series [8, 16–18]. However, there is still scepticism about the long-term benefits with regard to disease-free survival (DFS) and overall survival (OS). There is also no clarity on whether CME is indicated in every patient or in selected cases. There is growing consensus that CME is more beneficial in high-risk cancers such as poor differentiation, N1/N2 disease on staging scans, younger patients and lesions in the transverse colon [19].

The advantage of CME surgery in improving survival has to be balanced against the morbidity of the procedure. CME has been adopted more widely in the Korea, Japan and China; however, in the West, surgeons have remained more conservative. Higher patient BMI, lack of training and lower surgical volumes may be some of the factors responsible for this difference. Lack of standardised nomenclature and operative technique makes it even harder to compare the published data in this field.

The aim of this study is to describe the results of a standardised technique for CME surgery for colon cancer with the use of DaVinci robotic platform. We analysed the feasibility, safety and short-to-midterm results of robotic CME. The authors preferred a SMV first approach in this case series.

## Methods

This is retrospective cohort study of the patients having robotic CME surgery for colon cancer prospective collected data. Patients operated between July 2015 and September 2019 were included in the study. Robotic CME was selectively offered to patients with right-sided colon cancers and included cancers of the ascending colon, hepatic flexure and transverse colon. Caecal cancers were only included if they had N2 disease on preoperative staging. In the earlier phase of the learning curve, patients with high BMI (>35) and older than 75 years were excluded due to the potential risk of complications (Tables 1, 2, 3). All patients were discussed at the multidisciplinary meeting and were staged preoperatively with CT chest abdomen and pelvis scan and in selected cases abdominal ultrasound and MRI abdomen, particularly when the resection margins were threatened. 3D CT reconstructions were carried out and a planning meeting with the radiologist was standard practice to delineate the anatomy preoperatively.

**Table 1:** Patient demographics

Demographics	Number (n)	Percentage (%)
Total	77	
Male	34	44
Age median (range)	69 (34–89)	
BMI median (range)	26 (17–42)	
ASA 1–2	56	73
ASA 3–4	21	27
Previous abdominal surgery	25	32
Site of tumour		
Caecum	14	18.2
Ascending colon	24	31.2
Hepatic flexure	18	23.4
Transverse colon	21	27.3

**Table 2:** Operative data

Operating time median	180 (128–454) min	
Docking time median	10 (5-30) min	
Console time median	155 (120-350) min	
Blood loss (ml) median (range)	10 (10–50) ml	
Conversion	0	
Hospital stay median	5 (3–18) days	
30 days readmission	7	9%
30 days reoperation	2	3%

**Table 3:** Morbidity with Clavien–Dindo classification

Patients (n)	Morbidity	Management	Clavien–Dindo
3	Wound infection	Opening and packing of wound	I
1	Abdominal pain	Analgesics	I
3	Ileus	Nasogastric tube and TPN	II
1	Small bowel injury	Surgical Intervention	IIIb
1	Small bowel obstruction	Surgical intervention	IIIb

The variables analysed included patient demographics, intraoperative variables like operative time, blood loss, conversion rate, postoperative length of stay, complications, pathology status including R0 rate, T and N stages, local and distant recurrence after surgery and survival rates as shown in Table 2.

Patients were consented in detail about the procedure, risks and complications and expected outcomes. All cases were recorded and specimens photographed for audit and training purposes and reported by colorectal pathologists according to a standard protocol.

Post op ileus was defined as lack of bowel activity (flatus/ feaces) for more than 72 hours. Anastomotic leak was defined as a clinical or radiological defect in the anastomosis with signs of sepsis. Patients were followed up at 6 and 12 months and then annually for the first 5 years. A CT scan of chest abdomen and pelvis was performed annually. Recurrences were diagnosed on radiology and confirmed with histology where appropriate.

Statistical analysis was carried out using GraphPad Prism version 8.4.2 for Mac, GraphPad Software, La Jolla California USA, “www.graphpad.com”.

## Surgical Technique

**Fig. 1:**
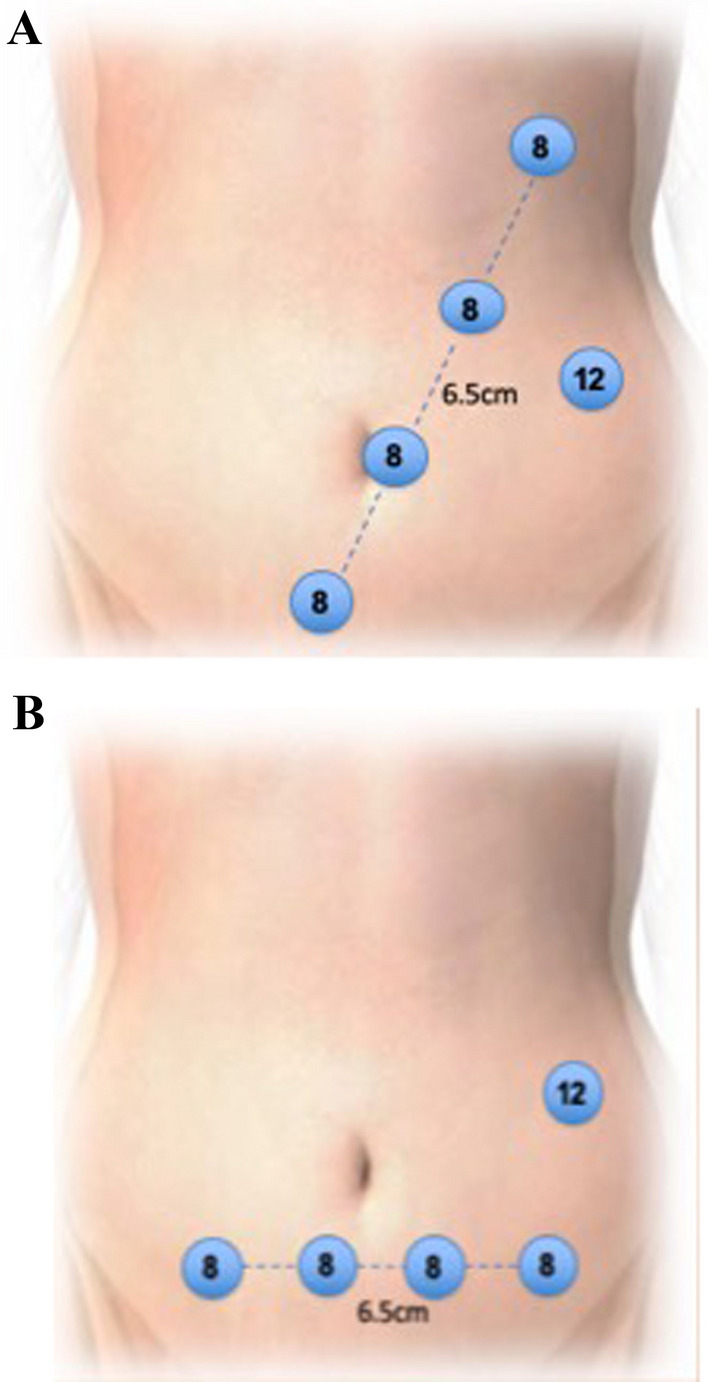
**A** and **B** Oblique and suprapubic port placement for Davinci system X

Patient is admitted on the day of surgery and receives a phosphate enema. In our institution, patient for right-sided cancer does not receive mechanical bowel prep prior to surgery. A dose of prophylactic antibiotics is given at induction of anaesthesia. Arterial phase CT was done prior to surgery to assess the arterial anatomy, and portal venous phase scanning is used for venous anatomical reconstruction. Da Vinci X system (Intuitive Surgical, California USA) is used in our institute. Patient is placed on an anti-slip mattress. Legs are placed in AV boots and in Lloyd Davis position. Operating table is placed in 10-degree Trendelenburg and 15-degree left tilt to move the small bowel away from the midline vessels. Linear port placement with 4 ports in an oblique line (as shown in Fig. 1A) is used for a standard CME; however, in our current set up, we prefer a suprapubic port placement as described below. Four robotic ports (Fig. 1B) placed in suprapubic area transversely 3cm away from bony prominences (anterior superior iliac spine) and 5 cm from symphysis pubis. At least 6 cm distance between each port is required to avoid clashing of the arms. Airseal port (assistant port) 12mm is used in left lumbar region 5–6cm away from the other ports.

**Fig. 2:**
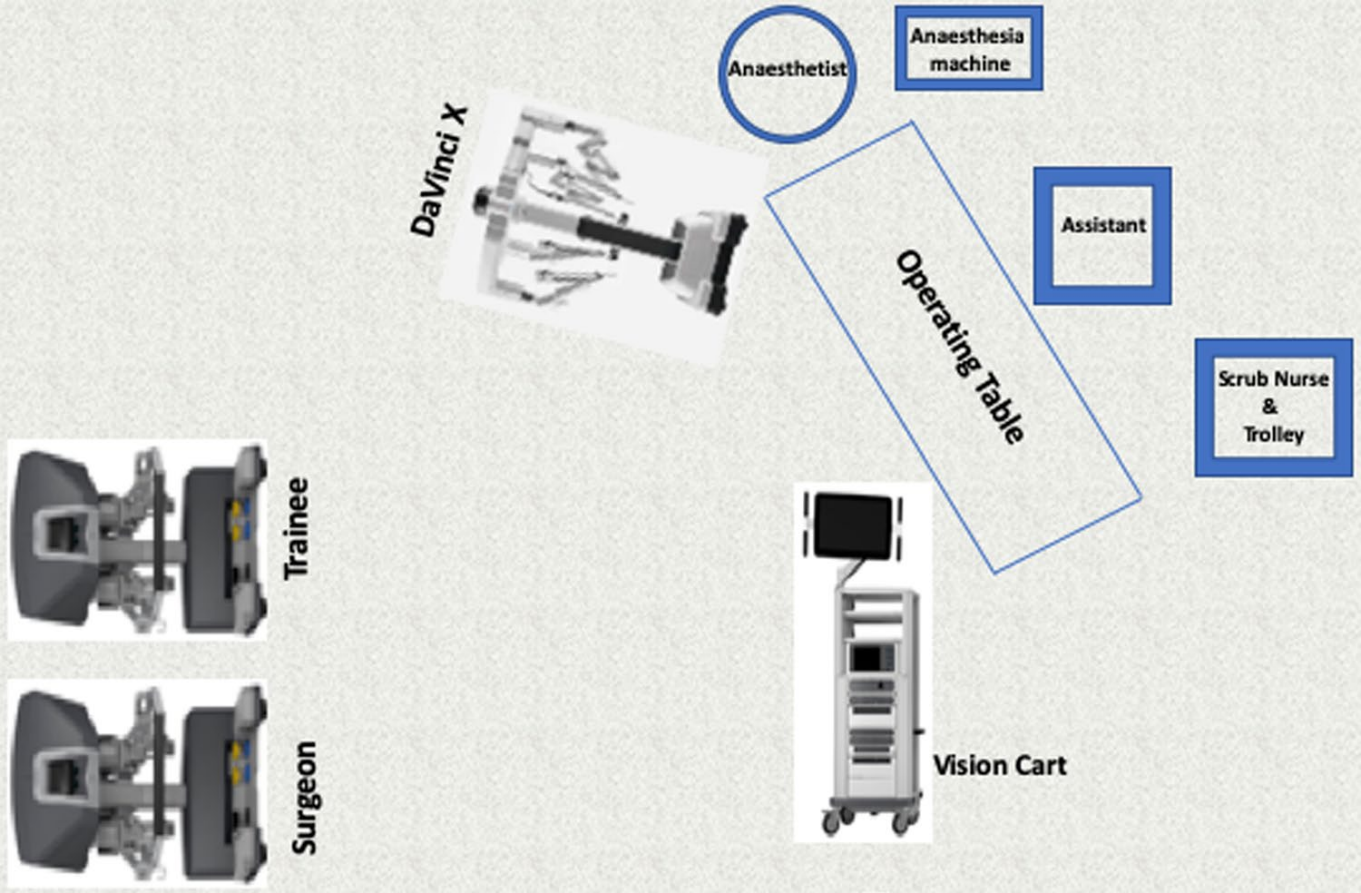
Operating room layout for CME

**Fig. 3:**
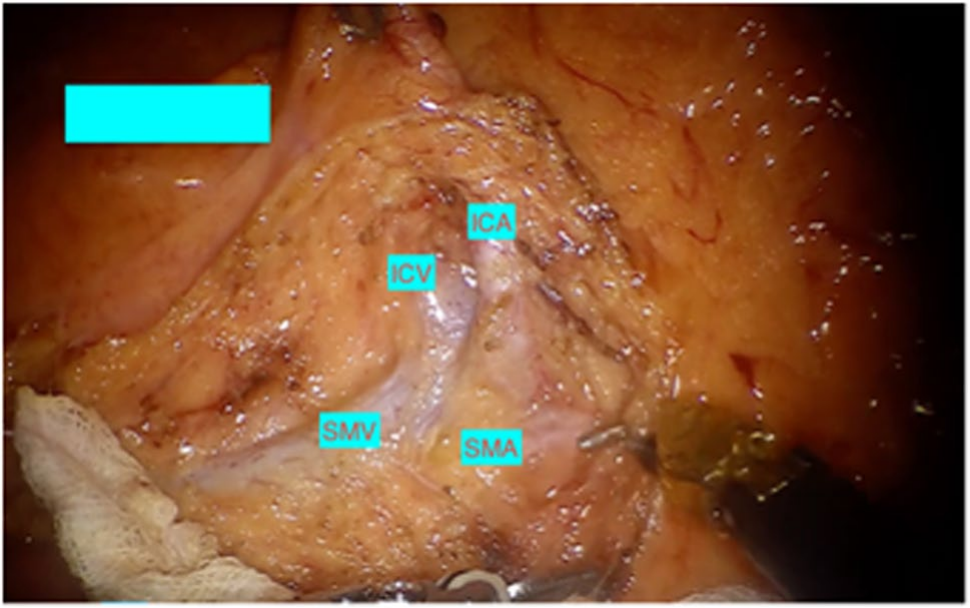
Origin of Ileocolic artery and vein

Docking is done from the right shoulder of the patient in case of suprapubic port placement. If oblique port placement is used, then the robot is docked from the right side of the patient for ascending colon cancers and moved towards the right shoulder for transverse colon cancers. The room layout is shown in Fig. 2. Initial dissection starts distally at SMV pedicle close to the terminal ileum. It is important to skeletonise and clear the fat in front of the SMV. In obese patients, we pioneered the use of intraoperative ultrasound to identify the SMV and SMA and facilitate safer dissection. Ileocolic vein (ICV) is divided first at its junction with the SMV (Fig. 3) followed by Ileocolic artery, which clipped at the right border of SMV.

**Fig. 4:**
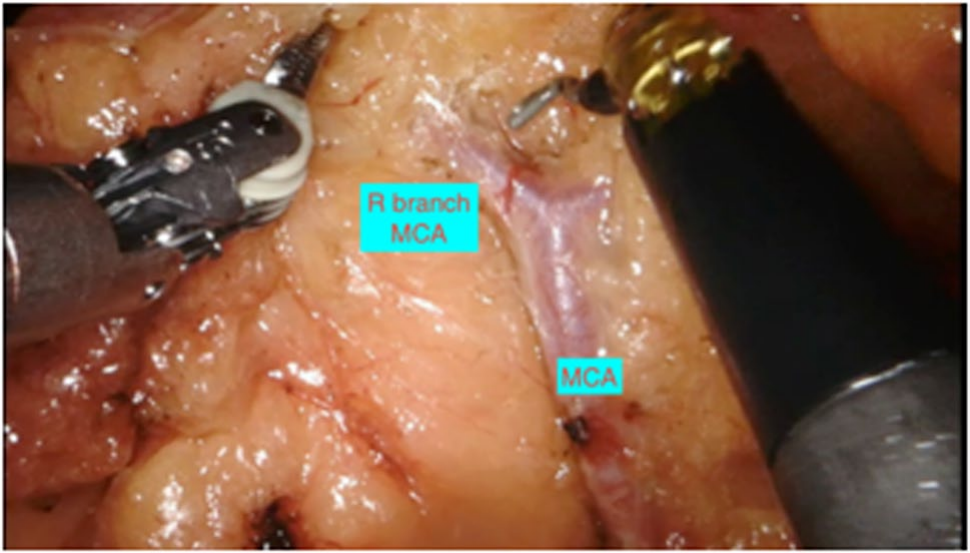
Middle colic artery dividing into right and left branches

Dissection continues over SMV cranially to identify the middle colic trunk and its division into right and left branches (Fig. 4), and for a standard right colectomy, the right branch of middle colic artery (MCA) is divided between clips.

**Fig. 5:**
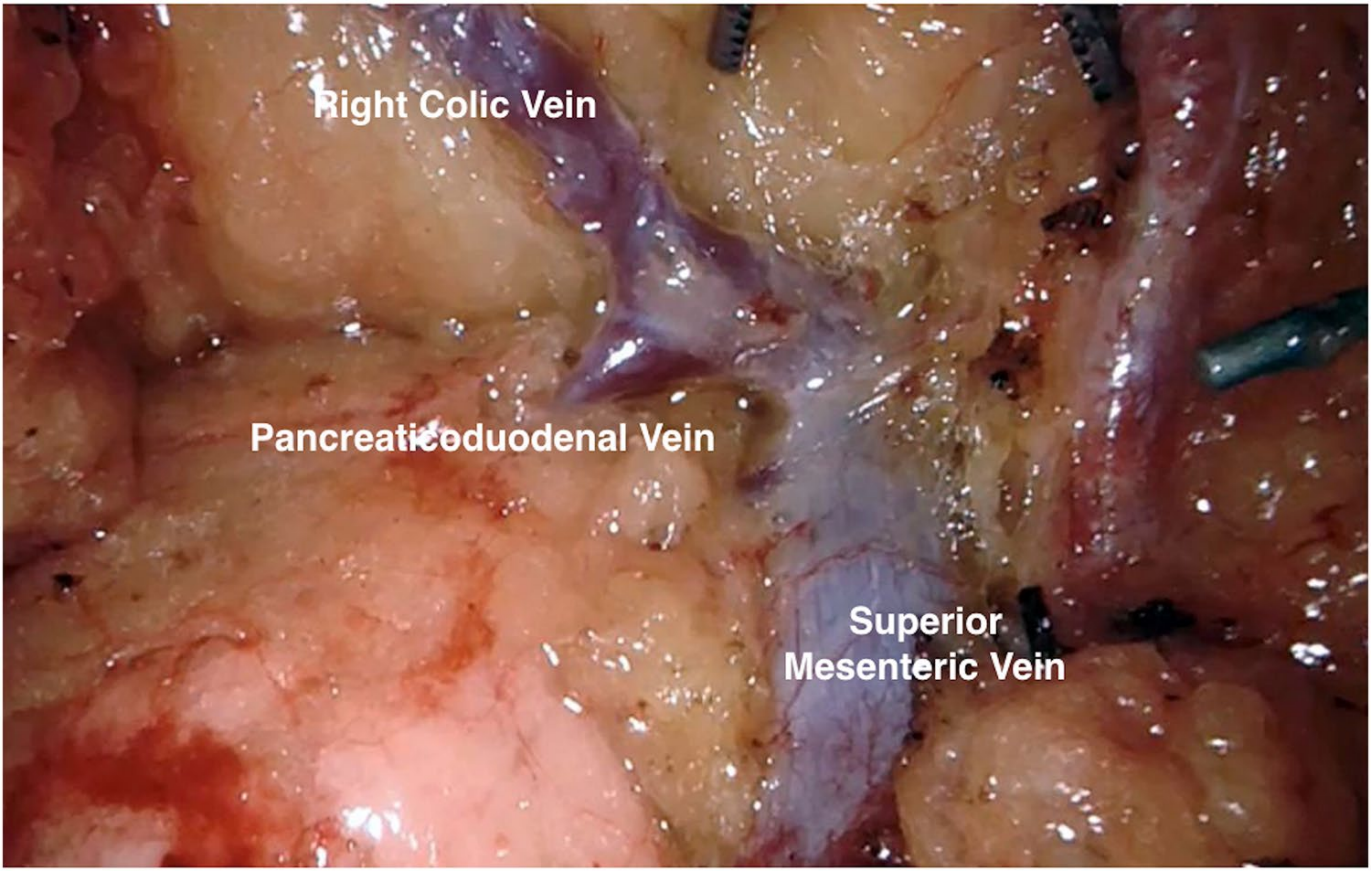
Trunk of Henle originating from SMV

For transverse colon cancers, an extended right hemicolectomy is carried out by dividing the middle colic artery and vein at its origin. Lastly, the Henle trunk is identified over the pancreas and although may have significant anatomical variations, careful identification and ligation of right colic vein, and preservation of pancreaticoduodenal veins, gastroepiploic vein and Henle trunk is preferred (Fig. 5).

After the central vascular division, medial-to-lateral dissection is carried out to separate the colonic mesentery from gerota’s fascia, duodenum and pancreas. Cranially, this plane is continued to gain entry into the lesser sac. Lateral peritoneal detachment is carried out up to the hepatic flexure. Gastrocolic omentum is divided starting from the level of falciform ligament and staying outside the gastroepiploic arcade. Colonic and small bowel mesentery is divided with robotic vessel sealer up to the intended point of transection. Intracorporeal division is done with the help of robotic SureForm 60mm stapler after assessment of the perfusion with ICG. Side-to-side isoperistaltic ileocolic anastomosis is performed with SureForm 60 mm stapler. Two stay stitches are placed one on either end of the bowel to facilitate this step. Common enterotomy (stapler ends) is closed with two-layer vicryl 3/0 stitch. Specimen is extracted through suprapubic pfannenstiel incision, using Alexis wound protector. A layered closure is performed with PDS and monocryl.

## Results

**Fig. 6:**
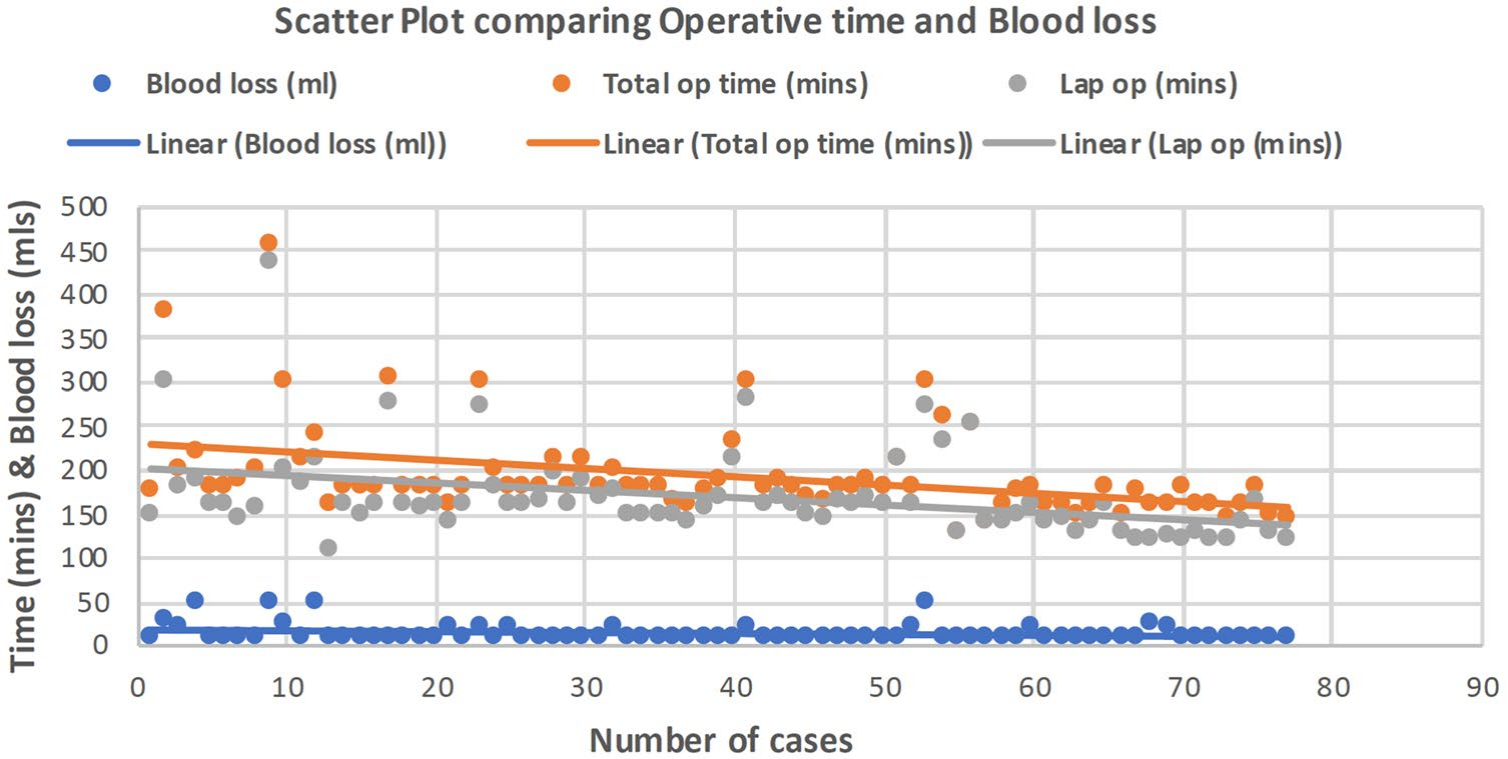
Blood loss and operating time scatter plot with the number of cases

Between July 2015 and September 2019, 77 patients underwent robotic CME for colon cancer. There were 34 males and 43 females with a median age of 69 (34–89) years. Patient demographics and location of cancers are described in Table 1. Median blood loss was 10 (10–50) ml (Fig. 6). 32% of patients had previous abdominal surgery and the median operating time was 180 (128–454) min (Fig. 6). There was no conversion in this case series. Median length of hospital stay was 5 (3–18) days (Table 2). Median follow up was 36 months.

## Clinical and oncological outcomes

Postoperative complications occurred in nine (11.6%) patients (Table 3). Two (3%) patients had reoperation within 30 days of surgery. One had small bowel injury and another patient had small bowel obstruction. In this group, three patients had wound infection and were treated with antibiotics and packing of wound. Three patients had ileus managed conservatively with total parenteral nutrition and one patient was admitted with abdominal pain settled with analgesics.

**Fig. 7:**
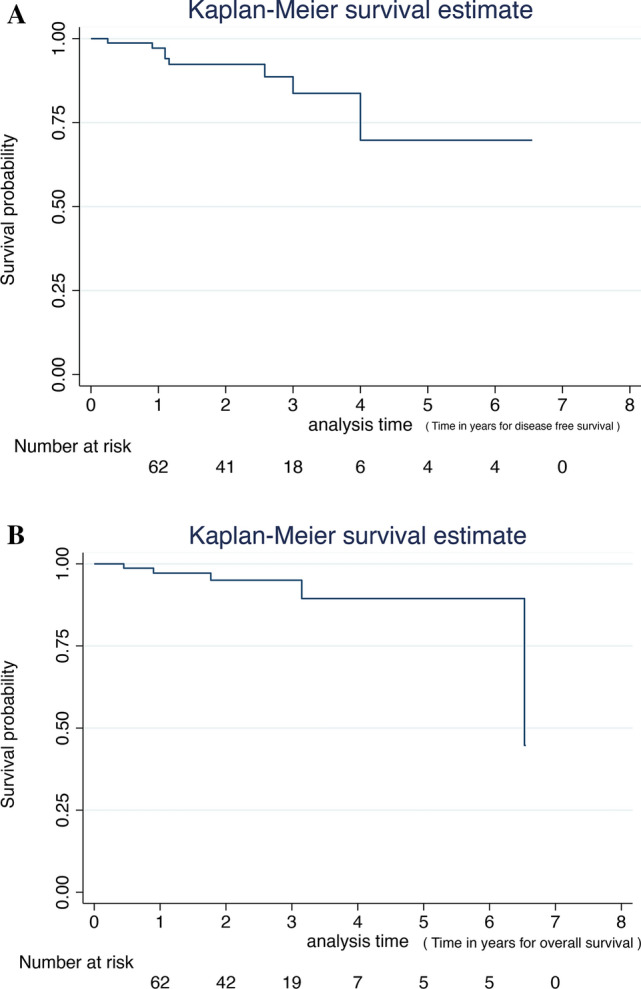
**a** Disease-free survival for robotic CME for colon cancer. **b** Overall survival for robotic CME for colon cancer

Median lymph node harvest was 30 (10–60). R0 resection was achieved in 96% of patients. The overall and disease-free survival was 94% (Table 3, Fig. 7a, b). Three (4%) patients had R1 resection. One of them had an involved distal resection margin 50 mm distal to the primary tumour, due to extensive sub-mucosal and lymphatic tumour invasion. One patient had a positive margin on abdominal wall and the other around para-nephric fat (both were T4 tumours).

**Fig. 8:**
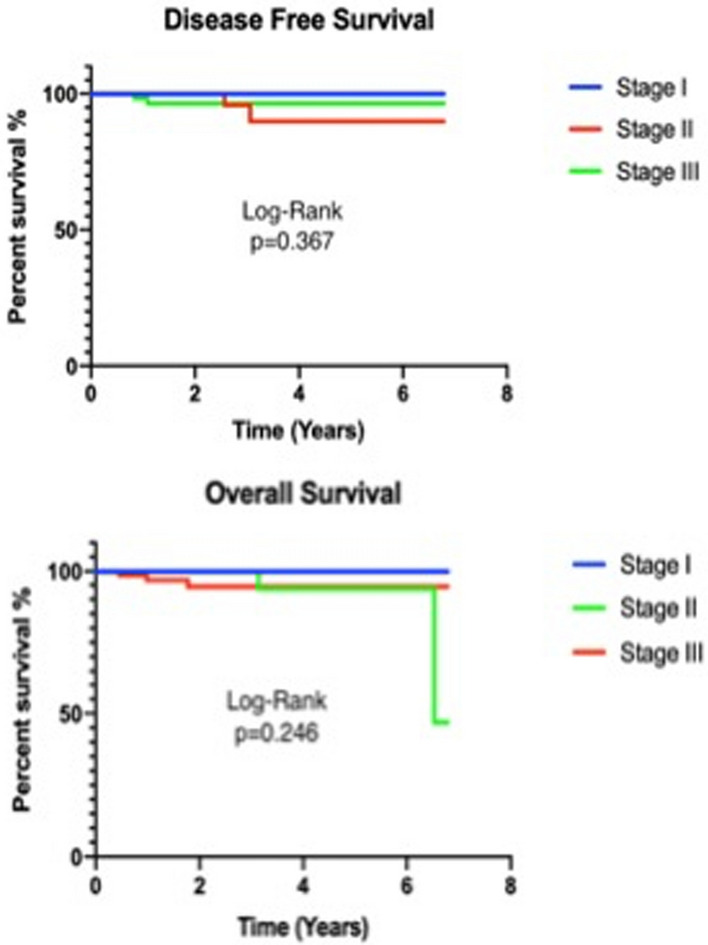
Overall and disease-free survival for robotic CME for colon cancer for stages I, II and III

Over two-thirds of the patients had T3 or T4 tumours, and N2 disease on histology was seen in 17% of patients (Table 4). There was one case of local recurrence seen in stage III disease and 4 (5%) distal recurrence in stage II and stage III. Three liver and one lung metastasis were recorded. There was no 30- or 90-day mortality. Overall survival at three years was 94%. Disease-free survival was 94%. Disease-free survival for stages I, II and III was 100%, 91.7% and 92%, respectively (Fig. 8). On comparison between stages I, II and III cancers, there was no statistical difference in DFS (p=0.367) and OS (p=0.246). In stage I, there was no recurrence and mortality.

**Table 4:** Oncological outcomes

	Number (n)	Percentage (%)
R0	74	96
R1	3	4
pT1–T2	17	22
pT3–T4	60	78
L.N harvest median (range)	30 (10–60)	
LN status		
N0	40	52
N1	24	31
N2	13	17
Local recurrence	1	1
Distal recurrence^§,*^	4	5

§ 3 liver, * 1 lung

## Discussion

The concept of CME is based on anatomical and precision surgery. This is likely to be linked with improved outcomes as has been shown in individual series [20–22]. The concern remains about the safety and feasibility in Caucasian population and the long-term oncological benefits. To date, no level I evidence exists to show the superiority of CME over standard hemicolectomy. There are a few studies in progress that may provide an answer in the future [23–26]. Meta-analysis of cohort studies has shown benefit of CME with reduced local recurrence and improved survival [27].

The principals of CME surgery are well established and include D3 lymphadenectomy with completely intact visceral peritoneum and central vascular ligation. Traditionally, this has been carried out as an open procedure. However, with the advancement in technology and experience in minimally invasive surgery, laparoscopic and robotic approaches have been used. There is no consensus on patient’s selection and criteria for CME surgery. However, most surgeons appear to offer CME surgery to younger patients with locally advanced tumours. The patients in our series were selected on the basis of their age, location of tumour and the nodal status on CT scans because we thought that these were the groups more likely to benefit from CME.

CME surgery takes longer than a standard right colectomy and this has been obvious with our results of median operating time of 180 min. This compares favourably with the published literature [28]. Operating time, however, is a composite measure of docking time, console time and team efficiency. We did notice that the surgical operating time improved after 30 cases and this was a combination of efficient docking and improved surgical experience (Fig. 7). The lymph node harvest has increased with CME surgery and the median count in our experience has been 30 (20–60), which is consistent with other published open CME series [8, 29, 30].

CME is often associated with prolonged ileus and longer length of stay due to the morbidity. Our median length of stay of 5 days supports that with precision surgery the complication rates are low and a quicker discharge can be achieved. This also supports the fact that dissection along the front of SMV does not lead to any significant nerve damage and avoids the incidence of prolonged ileus and bowel dysfunction. Intracorporeal anastomosis may have also contributed to the reduced ileus incidence by reducing the mobilisation of the transverse colon and less traction on gastrocolic omentum.

One of the fears of CME surgery is the increased morbidity and the risk of vascular and pancreatic injuries. [31]. In laparoscopy, the lack of internal triangulation to expose structures and the difficulty to safely operate around major vessels can result in increased morbidity of the procedure especially in obese patients. Robotic surgery has the potential to offset these limitations. In our series there were no cases of major vascular injury, chyle leak or pancreatic injury. The median blood loss was minimal and no conversion to open surgery was needed.

In our study, robotic CME was associated with 3-year DFS and OS of 94% for all patients, which favours very well with the Erlangen series of open CME [8]. There are only few studies to date that have reported short- or long-term oncologic outcomes for minimally invasive CME with type of procedure and length of follow up (Table 5).

**Table 5:** Comparison of oncologic results of Portsmouth series with published data

Comparison with other series	Patients (n)	Type of surgery	Type of approach	Mean FUP (Years)	DFS (%)	OS (%)
Portsmouth series	77	Right colectomy	Robotic	3	94%	94%
Spinoglio et al. [32]	100	Right colectomy	Robotic	4	91.40%	90.3
Cho et al. [33]	773	Right colectomy	Open and MIS	5	Open 82.9 MIS 82.8	Open 89.8 MIS 84
Bertelsen et al. [18]	364	Colectomy	Open and lap	4	85.8	74.9
Shin et al. [34]	168	Colectomy	Lap	4.8	88.3	89.6
Hohenberger et al. [8]	1329	Colectomy	Open	5	89.1	
Bokey et al. [20]	779	Colectomy	Open and lap	5	89.8	76.2

*CME* complete mesocolic excision, *MIS* minimally invasive surgery, *Lap* laparoscopic, *FUP* follow-up time, *DFS* disease-free survival, *DSS* disease-specific survival, *OS* overall survival

We favour robotic SMV first approach. A sub-ileal and lateral to medial approach can also be used for CME surgery; however, we believe that starting the procedure on SMV will facilitate the vascular ligation on its origin and this would make the procedure easier by having an early control of the main vessels. The use of intraoperative ultrasound to identify central vessels, the use of vessel sealer to divide the mesentery and robotic hem-o-lok clips application can all facilitate the procedure. ICG dye is useful in assessing the blood supply of the bowel especially during intracorporeal anastomosis when the marginal bleeding cannot be tested.

This procedure is, however, not for a novice because of its complexity, anatomical variations and handling of lymphovascular structures near central vessels. A surgeon should have extensive training in colonic surgery and good anatomical knowledge of pancreas, central vessels and gastrocolic area before embarking on CME surgery. The procedure can be further facilitated by familiarising with different surgical techniques including tips and tricks. Enrolment in to a training program with a set of proctored cases is likely to reduce the learning curve and the incidence of morbidity.

This is a retrospective series with a degree of selection bias; however, as mentioned earlier, younger patients with locally advanced disease were chosen to undergo CME surgery. It’ is a single-surgeon series and although the DFS and OS are good at 3 years, it will be important to review this with further follow up. The generalizability of this technique remains a question and the authors are in the process of starting a training program for CME and further longitudinal data will be needed to answer this question and provide insight into the learning curve for CME.

## Abbreviations

BMI: Body mass index
CME: Complete mesocolic excision
DFS: Disease-free survival
TME: Total mesorectal excision
Fig: Figure
ICA: Ileocolic artery
ICV: Ileocolic vein
ICG: Indocyanine green
MCA: Middle colic artery
MCV: Middle colic vein
SMV: Superior mesenteric vein
SMA: Superior mesenteric artery
OS: Overall survival

## References

[1] Lacy AM, García-Valdecasas JC, Delgado S, Castells A, Taurá P, Piqué JM, Visa J (2002). Laparoscopy-assisted colectomy versus open colectomy for treatment of non-metastatic colon cancer: a randomised trial. Lancet (London, England).

[2] Colon Cancer Laparoscopic or Open Resection Study Group, Buunen M, Veldkamp R, Hop WCJ, Kuhry E, Jeekel J, Haglind E, Påhlman L, Cuesta MA, Msika S, Morino M, Lacy A, Bonjer HJ (2009) Survival after laparoscopic surgery versus open surgery for colon cancer: long-term outcome of a randomised clinical trial. Lancet Oncol 10:44–52. 10.1016/S1470-2045(08)70310-3 10.1016/S1470-2045(08)70310-3 19071061

[3] Fleshman J, Sargent DJ, Green E, Anvari M, Stryker SJ, Beart RW, Hellinger M, Flanagan R, Peters W, Nelson H, Clinical Outcomes of Surgical Therapy Study Group (2007) Laparoscopic colectomy for cancer is not inferior to open surgery based on 5-year data from the COST Study Group trial. Ann Surg 246:655–62; discussion 662-4. 10.1097/sla.0b013e318155a762 10.1097/SLA.0b013e318155a762 17893502

[4] Heald RJ, Ryall RD (1986). Recurrence and survival after total mesorectal excision for rectal cancer. Lancet (London, England).

[5] Cecil TD, Sexton R, Moran BJ, Heald RJ (2004) Total mesorectal excision results in low local recurrence rates in lymph node-positive rectal cancer. Dis Colon Rectum 47:1145–9; discussion 1149-50. 10.1007/s10350-004-0086-6 10.1007/s10350-004-0086-6 15164243

[6] Wasmuth HH, Nestvold T, Røkke O (2008). Low local recurrence rates after rectal cancer resection with limited use of preoperative radiotherapy. Scand J Surg.

[7] Baran B, Mert Ozupek N, Yerli Tetik N, Acar E, Bekcioglu O, Baskin Y (2018). Difference Between Left-Sided and Right-Sided Colorectal Cancer: A Focused Review of Literature. Gastroenterol Res.

[8] Hohenberger W, Weber K, Matzel K, Papadopoulos T, Merkel S (2009) Standardized surgery for colonic cancer: complete mesocolic excision and central ligation–technical notes and outcome. Colorectal Dis 11:354–64; discussion 364-5. 10.1111/j.1463-1318.2008.01735.x 10.1111/j.1463-1318.2008.01735.x 19016817

[9] Zheng M, Ma J, Fingerhut A, Adamina MP, Atroschenko A, Bergamaschi R, Berho M, Boni L, Chadi SA, Chen WT-L, Delaney CP, Dapri G, Khatkov IE, Kim N-K, Kim S-H, Karachun A, Lomanto D, MacRae H, Milone M, Morino M, Remzi FH, Uranues S, Watanabe M, Wexner S (2018) Complete mesocolic excision for colonic cancer: Society for Translational Medicine expert consensus statement. Ann. Laparosc. Endosc. Surgery; Vol 3, No 8 (2018) Ann Laparosc Endosc Surg

[10] Hamzaoglu I, Ozben V, Sapci I, Aytac E, Aghayeva A, Bilgin IA, Bayraktar IE, Baca B, Karahasanoglu T (2018). “Top down no-touch” technique in robotic complete mesocolic excision for extended right hemicolectomy with intracorporeal anastomosis. Tech Coloproctol.

[11] Matsuda T, Iwasaki T, Mitsutsuji M, Hirata K, Maekawa Y, Tanaka T, Shimada E, Kakeji Y (2015). Cranial-to-caudal approach for radical lymph node dissection along the surgical trunk in laparoscopic right hemicolectomy. Surg Endosc.

[12] Schulte Am Esch J, Iosivan S-I, Steinfurth F, Mahdi A, Förster C, Wilkens L, Nasser A, Sarikaya H, Benhidjeb T, Krüger M (2019). A standardized suprapubic bottom-to-up approach in robotic right colectomy: technical and oncological advances for complete mesocolic excision (CME). BMC Surg.

[13] Yang Y, Malakorn S, Zafar SN, Nickerson TP, Sandhu L, Chang GJ (2019). Superior Mesenteric Vein-First Approach to Robotic Complete Mesocolic Excision for Right Colectomy: Technique and Preliminary Outcomes. Dis Colon Rectum.

[14] Spinoglio G, Bianchi PP, Marano A, Priora F, Lenti LM, Ravazzoni F, Petz W, Borin S, Ribero D, Formisano G, Bertani E (2018). Robotic versus laparoscopic right colectomy with complete mesocolic excision for the treatment of colon cancer: perioperative outcomes and 5-year survival in a consecutive series of 202 patients. Ann Surg Oncol.

[15] Agalianos C, Gouvas N, Dervenis C, Tsiaoussis J, Theodoropoulos G, Theodorou D, Zografos G, Xynos E (2017). Is complete mesocolic excision oncologically superior to conventional surgery for colon cancer? A retrospective comparative study. Ann Gastroenterol.

[16] Xie Y, Wang J, Hu L, Li H (2014). A meta-analysis of feasibility and safety in complete mesocolic excision for colon cancer. Zhonghua Wei Chang Wai Ke Za Zhi.

[17] Bae SU, Yang SY, Min BS (2019). Totally robotic modified complete mesocolic excision and central vascular ligation for right-sided colon cancer: technical feasibility and mid-term oncologic outcomes. Int J Colorectal Dis.

[18] Bertelsen CA, Neuenschwander AU, Jansen JE, Kirkegaard-Klitbo A, Tenma JR, Wilhelmsen M, Rasmussen LA, Jepsen L V, Kristensen B, Gögenur I, Copenhagen Complete Mesocolic Excision Study (COMES), Danish Colorectal Cancer Group (DCCG) (2016). Short-term outcomes after complete mesocolic excision compared with ‘conventional’ colonic cancer surgery. Br J Surg.

[19] Ouyang M, Luo Z, Wu J, Zhang W, Tang S, Lu Y, Hu W, Yao X (2019). Comparison of outcomes of complete mesocolic excision with conventional radical resection performed by laparoscopic approach for right colon cancer. Cancer Manag Res.

[20] Bokey L, Chapuis PH, Chan C, Stewart P, Rickard MJFX, Keshava A, Dent OF (2016). Long-term results following an anatomically based surgical technique for resection of colon cancer: a comparison with results from complete mesocolic excision. Colorectal Dis.

[21] Storli KE, Lygre KB, Iversen KB, Decap M, Eide GE (2017). Laparoscopic complete mesocolic excisions for colonic cancer in the last decade: Five-year survival in a single centre. World J Gastrointest Surg.

[22] Kotake K, Mizuguchi T, Moritani K, Wada O, Ozawa H, Oki I, Sugihara K (2014). Impact of D3 lymph node dissection on survival for patients with T3 and T4 colon cancer. Int J Colorectal Dis.

[23] Lu J-Y, Xu L, Xue H-D, Zhou W-X, Xu T, Qiu H-Z, Wu B, Lin G-L, Xiao Y (2016). The Radical Extent of lymphadenectomy - D2 dissection versus complete mesocolic excision of LAparoscopic Right Colectomy for right-sided colon cancer (RELARC) trial: study protocol for a randomized controlled trial. Trials.

[24] PIONEER Study: ichgcp.net/clinical-trials-registry/NCT03992599/

[25] Karachun A, Petrov A, Panaiotti L, Voschinin Y, Ovchinnikova T (2019). Protocol for a multicentre randomized clinical trial comparing oncological outcomes of D2 versus D3 lymph node dissection in colonic cancer (COLD trial). BJS open.

[26] Trial T-R (2019) International Prospective Observational Cohort Study for Optimal Bowel Resection Extent and Central Radicality for Colon Cancer (T-REX). NCT02938481. 10.1093/jjco/hyaa115 PMC7767979 33215206

[27] Killeen S, Mannion M, Devaney A, Winter DC (2014). Complete mesocolic resection and extended lymphadenectomy for colon cancer: a systematic review. Colorectal Dis.

[28] Ho ML, Chong C, Yeo SA, Ng CY (2019). Initial experience of laparoscopic right hemicolectomy with complete mesocolic excision in Singapore: a case series. Singapore Med J.

[29] West NP, Hohenberger W, Weber K, Perrakis A, Finan PJ, Quirke P (2010). Complete mesocolic excision with central vascular ligation produces an oncologically superior specimen compared with standard surgery for carcinoma of the colon. J Clin Oncol.

[30] Yamamoto S, Inomata M, Katayama H, Mizusawa J, Etoh T, Konishi F, Sugihara K, Watanabe M, Moriya Y, Kitano S, Japan Clinical Oncology Group Colorectal Cancer Study Group (2014). Short-term surgical outcomes from a randomized controlled trial to evaluate laparoscopic and open D3 dissection for stage II/III colon cancer: Japan Clinical Oncology Group Study JCOG 0404. Ann Surg.

[31] Koh FH, Tan K-K (2019). Complete mesocolic excision for colon cancer: is it worth it?. J Gastrointest Oncol.

[32] Spinoglio G, Marano A, Bianchi PP, Priora F, Lenti LM, Ravazzoni F, Formisano G (2016). Robotic right colectomy with modified complete mesocolic excision: long-term oncologic outcomes. Annals of Surgical Oncology.

[33] Cho MS, Baek SJ, Hur H, Soh Min B, Baik SH, Kyu Kim N (2015) Modified complete mesocolic excision with central vascular ligation for the treatment of right-sided colon cancer: long-term outcomes and prognostic factors. Ann Surg 261:708–715 10.1097/SLA.0000000000000831 25072438

[34] Shin JW, Amar AHY, Kim SH, Kwak JM, Baek SJ, Cho JS, Kim J (2014). Complete mesocolic excision with D3 lymph node dissection in laparoscopic colectomy for stages II and III colon cancer: long-term oncologic outcomes in 168 patients. Tech Coloproctol.

